# Epoxy-Thiol Systems Filled with Boron Nitride for High Thermal Conductivity Applications

**DOI:** 10.3390/polym10030340

**Published:** 2018-03-20

**Authors:** John M. Hutchinson, Frida Román, Adrià Folch

**Affiliations:** Departament de Màquines i Motors Tèrmics, ESEIAAT, Universitat Politècnica de Catalunya, C/Colom 11, 08222 Terrassa, Spain; roman@mmt.upc.edu (F.R.); afolch1@gmail.com (A.F.)

**Keywords:** epoxy, thiol, boron nitride, differential scanning calorimetry (DSC), thermal conductivity

## Abstract

An epoxy-thiol system filled with boron nitride (BN), in the form of 80 µm agglomerates, has been investigated with a view to achieving enhanced thermal conductivity. The effect of BN content on the cure reaction kinetics has been studied by differential scanning calorimetry (DSC) and the thermal conductivity of the cured samples has been measured by the transient hot bridge method. The heat of reaction and the glass transition temperature of the fully cured samples are both independent of the BN content, but the cure reaction kinetics is not: with increasing BN content, the reaction first advances and is then delayed, this behaviour being more pronounced than for the same system with 6 µm BN particles, investigated previously. This dependence on BN content is attributed to the effects of heat transfer, and the DSC results can be correlated with the thermal conductivity of the cured systems, which is found to increase with both BN content and BN particle size. For a given BN content, the values of thermal conductivity obtained are significantly higher than many others reported in the literature, and achieve a value of over 4.0 W/mK for a BN content of about 40 vol %.

## 1. Introduction

The design of modern microelectronic systems involves increasing miniaturisation and operation at higher frequencies than hitherto, resulting in increased power densities, and hence, greater risk of failure as a consequence of heat management problems. For example, it has been estimated that the failure rate of an electronic device doubles with every 10 °C increase in chip junction temperature [1], while for light emitting diodes (LEDs), there is a rule of thumb that every increase in operating temperature of 10% reduces the service life by 50% [2]. Thermal dissipation from such devices is therefore clearly of great importance. Heat transfer may take place by several mechanisms, namely conduction, convection, and radiation, but the most effective method is by conduction. The usual design involves the use of insulated metal substrates (IMS), in which the printed circuit layer (the heat source) is bonded to a metal substrate (the heat sink) by means of a dielectric layer, the properties of this layer being of prime importance in defining the performance of the IMS. The two principal properties required are electrical insulation and high thermal conductivity, which should be combined also with ease of processing (the dielectric layer is usually also the adhesive between the printed circuit layer and the metal substrate) and affordable cost.

One approach towards achieving these principal properties of the dielectric layer is to use an epoxy resin as the adhesive, and fill it with, typically, either boron nitride (BN) or aluminium nitride (AlN) particles to provide the pathways for heat conduction through the epoxy matrix. This approach has proved reasonably effective, and epoxy-based composites filled with these particles are the basis for some commercially available IMS systems, such as Tlam™ [3] from Laird Technologies and T-Clad [4] from Bergquist, which have thermal conductivities typically around 3.0 W/mK in comparison with about 0.2 W/mK for conventional printed circuit board technology.

**Figure 1:**
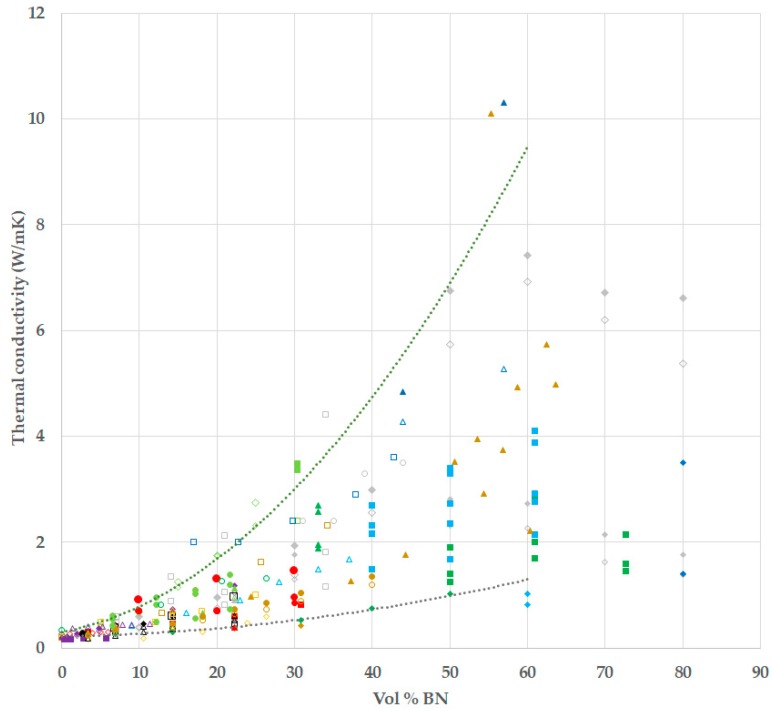
Dependence of thermal conductivity of epoxy-boron nitride (BN) composites on vol % of BN. Data taken from the literature, with the BN particles size(s) or range given in microns, larger symbols for larger particles. Diamonds: grey [5] 3.6; black [6] 0.07, 5; red [7] 0.06; orange [8] 0.4; brown [9] 0.5; light green [10] 13, 25; dark green [11] 0.9; light blue [12] 1, 5; dark blue [13] 6, 18; purple [14] 3–5. Squares: grey [15] 10; black [16] <1, 5–10; red [17] 2; orange [18] 2; brown [19] 6; light green [20] 5–10; dark green [21]; light blue [22] 1–12; dark blue [23] 50; purple [24] 0.004–0.040. Triangles: grey [25] 4–8; black [26] 1; red [27]; orange [28] 1, 5; brown [29] 3–5; light green [30]; dark green [31] 30; light blue [32] 30; dark blue [33] 5–11; purple [34] 100. Circles: grey [35] 10–30; black [36] 3; red [37] 0.053, 0.15, 4; orange [38] 0.07, 7; brown [39] 0.5; light green [40] 0.07, 7; dark green [41] 7–15. Filled symbols represent composites made with BN particles surface treated and/or with coupling agent; open symbols for untreated particles and without coupling agent. Data points in colour can be seen in the on-line version of this paper. Dotted lines are upper and lower trend curves, drawn to guide the eye.

Both higher and lower values of thermal conductivity than that presented by the above commercial systems have been reported in the literature for epoxy-BN and epoxy-AlN composites; we provide here a brief review of the results obtained for epoxy-BN composites, this being the system investigated in the present work. It is almost universally accepted that the thermal conductivity of epoxy-BN composites increases with BN content, at least up to about 60 volume percent (vol %), and that this increase becomes more marked the higher the BN content, giving an upward curvature in a plot of thermal conductivity as a function of the vol % of BN. A compilation of literature results using the search terms “epoxy” and “boron nitride” and “thermal conductivity” is presented in Figure 1, in which this behaviour can be seen.

It is evident that the same general trend is observed for all these results, but also that there is considerable scatter, while nearly all the results fall within the upper and lower trend curves indicated in the figure. There are several possible reasons for this scatter, related to the way in which these epoxy-BN composites are fabricated. The most important factors are: (i) the interface between particles and matrix, for which, on the one hand, the particles may be surface modified or a coupling agent may be used, or, on the other hand, the particles may be untreated; and (ii) the size of the particles, or the distribution of sizes, or even the combination of particles of different sizes.

In Figure 1, the composites for which either surface treated particles or a coupling agent were used are indicated as filled points, while composites with untreated particles and no coupling agent are indicated as open points. There is no obvious tendency for the filled points to approach the upper trend curve and for the open points to approach the lower trend curve, as would occur if there were a clear benefit in modifying the BN surfaces or in using a coupling agent. Nevertheless, some authors report a significant, if not always dramatic, increase in thermal conductivity when a coupling agent is used [5,12,14,20,22,30,31,33,39], the increase ranging from 12% to 45%. On the other hand, Kim and Kim [21] report an increase for –OH treatment, but a decrease for silane treatment, while other authors [6,9,11,28,29,36] report only a small increase, often less than 5%, as a result of surface treatment, at least until very high BN concentrations [6].

As regards the effect of BN particle size, the general trend is for the thermal conductivity to increase as the particle size increases, for the same vol % of BN [5,6,10,13,16,22,37,40]. Nevertheless, there are some exceptions: Hong et al. [12] report a higher value of thermal conductivity for 1 µm particles compared with 5 µm particles in a composite with 60 vol % BN; Permal et al. [27] find no difference between samples with 30 wt % BN fabricated with 1 and 5 µm particles; and Wattanakul et al. [32] report that sonication reduces the agglomerate size and increases the thermal conductivity, though this may also reflect the effect of particle dispersion in the epoxy matrix. In addition, the effect of particle size may be influenced also by the effect of particle shape. For example, Gaska et al. [10] find that larger particles, namely 25 µm spherical agglomerates, have a higher thermal conductivity than smaller particles, namely 13 µm platelets, but the different shapes of these two particles may have an effect. Likewise, the increase in thermal conductivity with increasing BN particle size reported by Huang et al. [16] may be affected by the different shapes of the particles, the smaller particles (1 µm) being spherical, and the larger (5 to 10 µm) ones being flakes, though this shape effect would be the opposite of that implied by the results of Gaska et al. [10].

The achievement of optimum thermal conductivity from these epoxy-BN composites clearly requires a better understanding of the effects of the various parameters involved. The aim of the present work is to extend our earlier study of epoxy-BN composites [19], in which two different epoxy systems as the matrix material were compared, both of them filled with 6 µm BN particles: epoxy-thiol initiated by an imidazole, and epoxy-diamine. It was found that the thermal conductivity of epoxy-BN composites based upon the epoxy-thiol system was significantly higher than that for the epoxy-diamine system for the same vol % BN, and this was attributed to an improved interface between matrix and particles in the epoxy-thiol system, as a consequence of a Lewis acid-base interaction between the boron in the particles and the sulphur in the thiol cross-linking agent. The enhanced thermal conductivity in the epoxy-thiol system was also mirrored in the cure kinetics, monitored by differential scanning calorimetry (DSC), where systematic effects were observed as a function of the BN content, and a systematic difference was observed between the two systems. In this respect, the DSC cure kinetics gave a clear indication of the likelihood of obtaining high thermal conductivity in any given epoxy-BN composite. Consequently, in the present work we propose to investigate the effect of BN particle size, including the use of hybrids with particles of different sizes, on both the cure kinetics and thermal conductivity of epoxy-BN composites based upon the epoxy-thiol system.

## 2. Materials and Methods

### 2.1. Materials

The epoxy resin used was diglycidyl ether of bisphenol-A, DGEBA (Araldite GY 240, Huntsman Advanced Materials, Salt Lake City, Utah, USA, 182 g/eq, density 1.17 g/cm^3^), with a thiol, pentaerythritol tetrakis (3-mercaptopropionate) (Sigma-Aldrich, Saint Louis, MO, USA, 488.66 g/mol, density 1.28 g/cm^3^) as the cross-linking agent. In order to initiate the cross-linking reaction of the epoxy with the thiol a latent initiator was used, encapsulated imidazole LC-80 (Technicure, A&C Catalysts, Linden, NJ, USA) in the form of powder. Different grades of BN filler were used: hexagonal plate-like particles with average particle sizes of 6 and 2 μm, and approximately spherical agglomerates of platelets, with an average particle size of 80 μm. The 6 μm particles were kindly provided by Benmayor S.A. (Barcelona, Spain), and the 80 μm agglomerates (PCTL5MHF) and 2 μm platelets (PCTP2) were obtained from Saint Gobain Boron Nitride (Amherst, NY, USA); all these BN particles were in the form of white powders and were used as received, without any surface treatment.

The different epoxy-BN composite materials were prepared as follows. The initiator, the smallest component and in a proportion of 2.0 parts per hundred resin (phr), was mixed with the thiol, in an amount calculated to maintain a stoichiometric ratio with the epoxy resin (approximately 60:40 epoxy/thiol by mass). The epoxy resin was then added, mixed thoroughly, and finally the BN filler was added in the proportion required, which ranged from 10% to 60% by mass, with respect to the combined mass of the BN and epoxy. All these components were mixed manually for about 10 min, until the appearance was homogenous, whereupon the mixture was placed in a vacuum chamber to degas, at room temperature and at a pressure of less than 26 hPa, for about 15 min.

Epoxy-BN composite simple mixtures were prepared in this way with the 80 μm particles alone, while hybrids were also prepared with a combination of particle sizes: 80 and 2 μm; 80 and 6 μm. The purpose was to use the smaller platelets to fill the spaces between the larger spherical agglomerates. For both hybrid systems, the mass of the smaller particles was one third of the mass of the 80 μm agglomerates, these last being added in the same proportions as for the simple mixtures.

A complete description of all the compositions used is given in Table 1. To make an approximate calculation of the volume percentages assuming additivity of volumes, the manufacturers’ values for the densities of the epoxy resin and the thiol, as given above, were used, while the density of BN was taken as 2.1 g/cm^3^.

### 2.2. Methods

#### 2.2.1. Differential Scanning Calorimetry (DSC)

Differential scanning calorimetry (DSC) was used, in both isothermal and non-isothermal (constant heating rate) modes, in order to monitor the cure reaction kinetics. In each case, a small sample of about 10 mg of the uncured epoxy-BN composite mixture was carefully weighed (Mettler Toledo analytical balance, AE 163, Greifensee, Switzerland, reproducibility 0.02 mg) into an aluminium capsule, crimped with a lid. The DSC experiments were performed using a conventional DSC (DSC821e, Mettler Toledo) equipped with a robot sample handler and intracooler (Haake EK90/MT, Vreden, Germany), with a dry nitrogen gas flow of 50 mL/min. The data evaluation was performed with the STAR^e^ software (Mettler Toledo, Greifensee, Switzerland), and the DSC was calibrated for both heat flow and temperature using indium.

For the isothermal cure experiments, the sample was introduced by the robot into the DSC furnace, which had previously been heated to the required isothermal cure temperature (60, 70 or 80 °C), and the heat flow was then measured as a function of time until the cure reaction was complete; the cure times were 200 min at 60 °C, 120 min at 70 °C, and 35 min at 80 °C. For the non-isothermal cure experiments, the sample was introduced by the robot into the DSC furnace at room temperature, cooled at 20 K/min to −65 °C, and then heated at the required constant rate (2, 5 or 10 K/min) to 200 °C.

The cure kinetics is characterised by the time, *t*_p_, at which the maximum heat flow occurs in isothermal cure, or by the temperature, *T*_p_, at which the heat flow is a maximum in non-isothermal cure, and in both experiments, by the exothermic heat of reaction, ∆*H*, obtained from the area under the cure curve. Additionally, the non-isothermal experiment allowed the determination of the glass transition temperature of the uncured mixture, *T*_g0_, to be obtained, occurring at a temperature well below that at which the cure reaction starts; this serves as a check on the reproducibility of the measurements, as it should remain constant for a stoichiometric epoxy-thiol system independent of the BN content.

After both the isothermal and non-isothermal cure experiments, a second scan (non-isothermal, at 10 K/min) was made to determine the final glass transition temperature, *T*_g∞_, of the fully cured sample and, for the isothermally cured samples, to check that there was no residual cure.

#### 2.2.2. Density Measurements

Epoxy-BN composite samples for the measurement of density were prepared by casting the uncured mixture in a silicone mould approximately 3 mm × 7 mm × 10 mm, which was then cured isothermally at 70 °C in an air-circulating oven for 3 h. The density was determined by Archimedes principle. The sample was first weighed in air (*m*_air_) at room temperature, and then when immersed fully in ethanol (*m*_eth_), suspended by a fine thread. The density of the sample, ρ_s_, is calculated from the density of ethanol, ρ_eth_, for which tabulated values are available as a function of the measured room temperature, by the equation:

ρ_s_ = *m*_air_/(*m*_air_ – *m*_eth_) × ρ_eth_ (1)

#### 2.2.3. Thermal Conductivity Measurements

The measurements of the thermal conductivity were made using the transient hot bridge method (Linseis, GmbH, THB-100, Selb, Germany) and a Kapton Hot Point sensor (Linseis) calibrated with polymethyl methacrylate (PMMA), borosilicate crown glass, marble, a Ti-Al alloy, and titanium, covering a range of thermal conductivities from 0.2 W/mK to above 10 W/mK. The epoxy-BN composite samples for the measurement of thermal conductivity were prepared by casting the uncured epoxy-BN mixture into silicone moulds, 10 mm × 40 mm × 4 mm. Two samples of any given composition were used for these measurements; the surfaces (10 mm × 40 mm faces) of each sample were carefully polished manually using emery paper (120, 400, and 600 grit size, sequentially), in order to give flat and smooth surfaces for contact with the Kapton sensor. The sensor was clamped between the two flat faces of the two samples, using a manual screw-actuated press. 

The THB-100 instrument applies a controlled heating power, typically 50 mW, to the sensor, which measures the resulting temperature change in the sample as a function of time; the higher the thermal conductivity of the sample, the more rapidly the heat is dissipated, and the smaller the corresponding temperature rise, Δ*T*, in the sample. The analysis of the heat flow under these circumstances [42] shows that a plot of Δ*T* as a function of the inverse square root of the time, 1/√*t*, is linear, and that the thermal conductivity, λ, can be determined from the extrapolated value of Δ*T* at infinite time (1/√*t* = 0). 

## 3. Results and Discussion

### 3.1. Differential Scanning Calorimetry (DSC)

**Figure 2:**
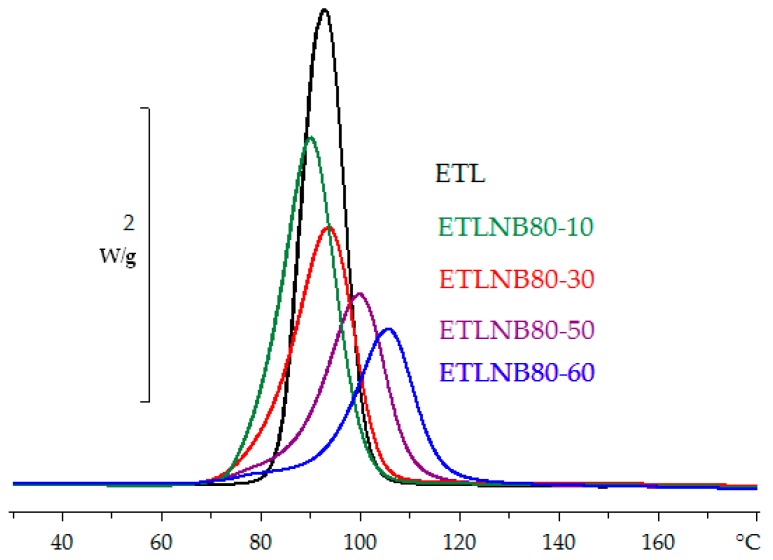
Non-isothermal DSC cure curves for epoxy-thiol system without BN (ETL) (black), ETLBN80-10 (green), ETLBN80-30 (red), ETLBN80-50 (purple), and ETLBN80-60 (blue) at the heating rate of 5 K/min. Exothermic direction is upward. The peak height decreases with increasing BN content.

Typical non-isothermal DSC cure curves for the epoxy-thiol system without BN (ETL) and for the simple composite system ETLBN80 with 80 μm BN agglomerates at a heating rate of 5 K/min and for the different BN contents, from 3.7 to 34.2 vol %, are shown in Figure 2. Only the temperature region in which the exothermic reaction occurs is shown here; at lower temperature, however, the glass transition of the uncured mixture appears at a temperature *T*_g0_ ≅ −37.2 °C, which is essentially independent of heating rate and of BN content, as can be seen from the results collected in Table 2, confirming the consistent stoichiometry of the epoxy-thiol mixtures. The decrease in the peak height and area as the BN content increases is a consequence of the decreasing proportion of epoxy resin, the heat flow in Figure 2 being normalised with respect to the total sample mass. When the exothermic heat of reaction, ∆*H*, is calculated per epoxy equivalent (ee), though, there is no significant variation with the BN content, as can be seen in Table 2; the average value is 130 ± 2 kJ/ee. Likewise, the glass transition temperature of the fully cured system, *T*_g∞_, is essentially independent of both heating rate and BN content, with an average value of 54.2 ± 1.5 °C (Table 2). The observation that both ∆*H* and *T*_g∞_ remain invariant suggests that the network structure of the cured system is independent of the BN content.

**Figure 3:**
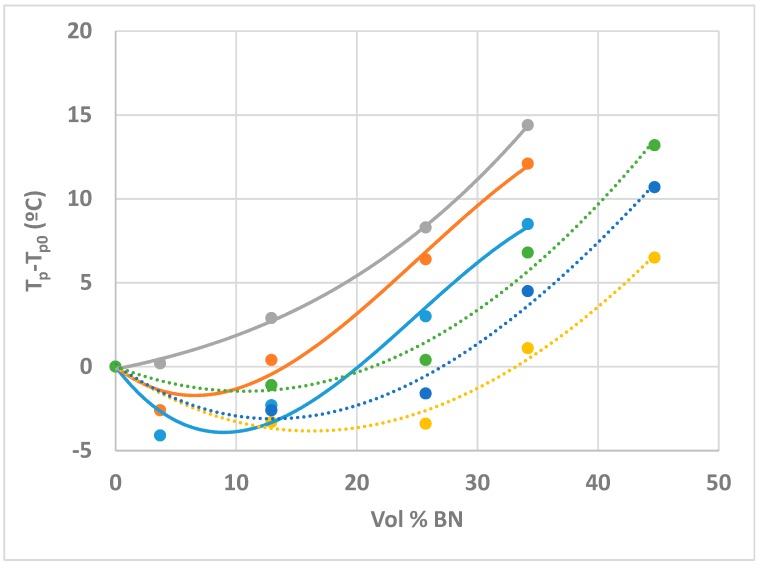
Plot of *T*_p_ − *T*_p0_ as a function of vol % BN for the systems ETLBN80 and ETLBN6, respectively, at the heating rates of 2 (dark blue, purple), 5 (red, light blue), and 10 K/min (green, orange). The curves are drawn to guide the eye: full lines for ETLBN80, dotted lines for ETLBN6.

On the other hand, it can be seen that a major effect of increasing the BN content is to shift the peak exotherm to higher temperatures. In fact, there is an initial decrease in the peak temperature before the monotonic increase with BN content, this decrease being even more pronounced at the slower heating rate of 2 K/min, as can be seen from the values listed in Table 2. This effect is more easily appreciated from a plot of *T*_p_ − *T*_p0_ as a function of BN content, shown in Figure 3 for the three heating rates, β, used here, where *T*_p0_ is the peak temperature for the ETL system without any filler.

A very similar dependence of the peak exotherm temperature on the BN content was reported earlier by ourselves [19] for the same epoxy-thiol system filled with BN platelets of 6 µm average size (system ETLBN6). The difference is that the ETLBN80 system presents this behaviour at lower BN contents than does ETLBN6, as can be seen in Figure 3. In the light of the observation above, that the heat of reaction and the glass transition temperature of the fully cured system are invariant, and hence that the cured network structure is independent of the BN content, we interpret the effect of the BN content on the cure kinetics, as demonstrated by Figure 3, as a physical effect, rather than a chemical one. This physical effect is considered to be related to the effect of the BN content on the thermal conductivity of the sample, which influences the rate of heat transfer into and out of the sample during the exothermic curing reaction.

**Figure 4:**
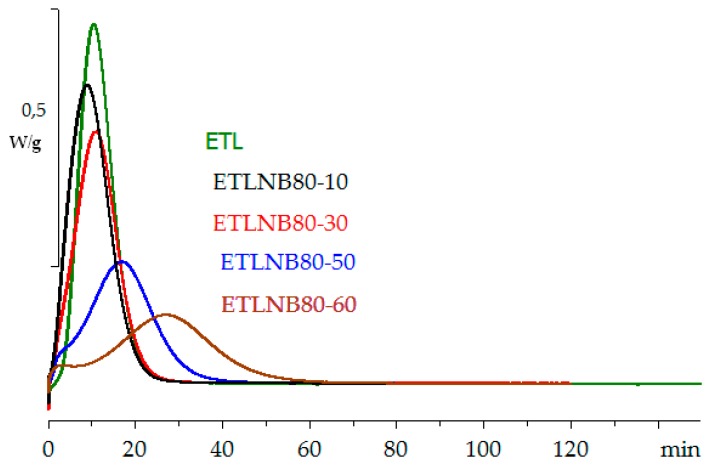
Isothermal DSC cure curves for ETL (green), ETLBN80-10 (black), ETLBN80-30 (red), ETLBN80-50 (blue), and ETLBN80-60 (brown) at 70 °C. Exothermic direction is upward. The peak height decreases with increasing BN content.

The same kind of dependence of cure kinetics on BN content is observed also for the isothermal cure reaction. Figure 4 shows the isothermal cure curves for the ETLBN80 system, for BN contents from 0 to 34.2 vol % and for a cure temperature of 70 °C. The decrease in the peak height and peak area with increasing BN content reflects the decreasing amount of reactive species. However, in the same way as for the non-isothermal cure results, when the heat of reaction, ∆*H*, is calculated per epoxy equivalent, it is found to be independent of the BN content, and of the isothermal cure temperature. Likewise, from the second (non-isothermal) scan after the isothermal cure, in which no residual reaction is observed, the glass transition temperature of the fully cured system, *T*_g∞_, is also found to be independent of both BN content and cure temperature. The values of both ∆*H* and *T*_g∞_ for isothermal cure are collected in Table 3.

**Figure 5:**
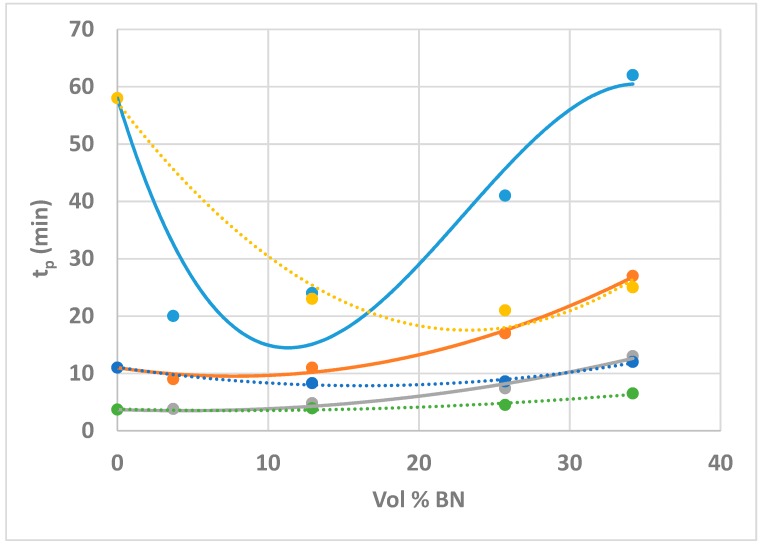
Plot of *t*_p_ as a function of vol % BN for the systems ETLBN80 and ETLBN6, respectively, at the isothermal cure temperatures of 60 °C (dark blue, purple), 70 °C (red, light blue), and 80 °C (green, orange). The curves are drawn to guide the eye: full lines for ETLBN80, dotted lines for ETLBN6.

Once again, therefore, it can be concluded that the cured epoxy-thiol network structure does not depend on the BN content, whereas the cure kinetics does. This is evident from the variation, with increasing BN content, of the time, *t*_p_, at which the peak exothermic heat flow occurs at each isothermal cure temperature, as illustrated in Figure 5. Here, it can be seen that there is an initial decrease in *t*_p_ with increasing BN content, indicative of an initial acceleration of the reaction, followed by an increase in *t*_p_ at higher BN contents, and that this decrease is most pronounced for the lowest cure temperature of 60 °C. Very similar results were obtained earlier for the ETLBN6 system [19], also included in Figure 5, the difference between the two systems being again that the effect of the BN content is more marked for the 80 µm agglomerates than for the 6 µm platelets.

These results lead to two important conclusions. First, we conclude that the addition of BN particles to the epoxy-thiol system results in a physical effect which modifies the cure reaction kinetics; this effect is believed to be related to the heat transfer into and out of the sample, and hence to be related to the thermal conductivity of the sample. The second conclusion is derived from the observation that the effect is more pronounced for the larger 80 µm agglomerates than it is for the smaller 6 µm platelets. The thermal conductivity is increased by creating pathways for heat transfer between the BN particles, but the main resistance to heat flow occurs at the interfaces between the BN particles and the epoxy matrix; it is for this reason that surface treatment of the BN particles generally leads to improved thermal conductivity, as it reduces the interfacial thermal resistance. In this respect, for the same vol % of BN particles, there will be fewer interfaces, the larger are the particles. We conclude that the more marked dependence on BN content found for the cure kinetics of the 80 µm agglomerates is a direct consequence of the effect of heat transfer, and hence we predict a higher thermal conductivity for the ETLBN80 system.

**Figure 6:**
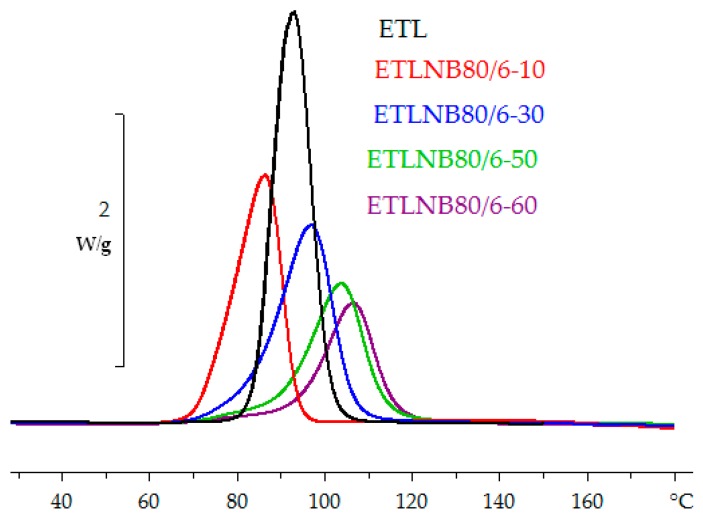
Non-isothermal DSC cure curves for ETL (black), ETLBN80/6-10 (red), ETLBN80/6-30 (blue), ETLBN80/6-50 (green), and ETLBN80/6-60 (purple) at the heating rate of 5 K/min. Exothermic direction is upward. The peak height decreases with increasing BN content.

**Figure 7:**
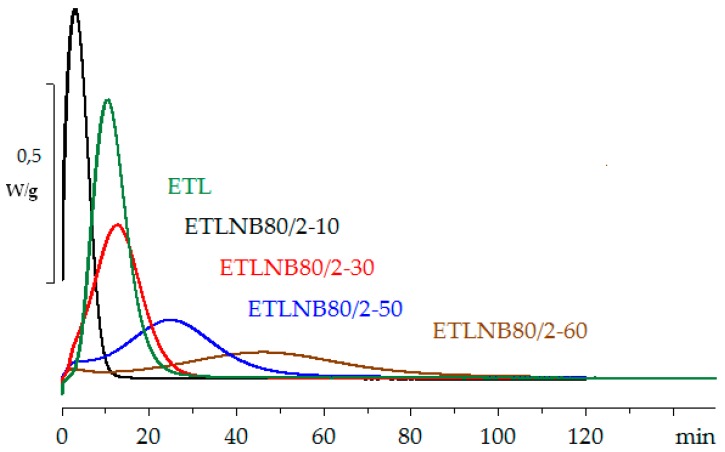
Isothermal DSC cure curves for ETL (green), ETLBN80/2-10 (black), ETLBN80/2-30 (red), ETLBN80/2-50 (blue), and ETLBN80/2-60 (brown) at 70 °C. Exothermic direction is upward. The peak height decreases with increasing BN content, except for ETLBN80/2-10.

Furthermore, this effect on the cure kinetics, either non-isothermal or isothermal, leads to the peak exothermic heat flow occurring at higher temperatures or at longer times, respectively, as the BN content increases. One would expect this trend to continue as the BN content, and hence the thermal conductivity, is further increased. However, there is a practical limit to the BN content when the simple mixing procedures used here are adopted, because the mixture becomes a very stiff paste at volume percentages greater than about 34%. One possibility to increase the vol % of BN, and hence increase the thermal conductivity, is through the use of hybrids in which particles of different sizes or types are combined, with the smaller particles filling the spaces between the larger, approximately spherical, agglomerates. The effect of hybrid systems on the thermal conductivity has been reported by several authors [10,13,17,18,23,27,30,40]. Two such hybrids have been used here: ETLBN80/6 and ETLBN80/2, in which 6 µm and 2 µm platelets, respectively, are included with the 80 µm agglomerates, as detailed in Table 1. Typical examples of the non-isothermal and isothermal cure curves, for ETLBN80/6 at 5 K/min and ETLBN80/2 at 70 °C, are shown in Figure 6 and Figure 7, respectively.

**Figure 8:**
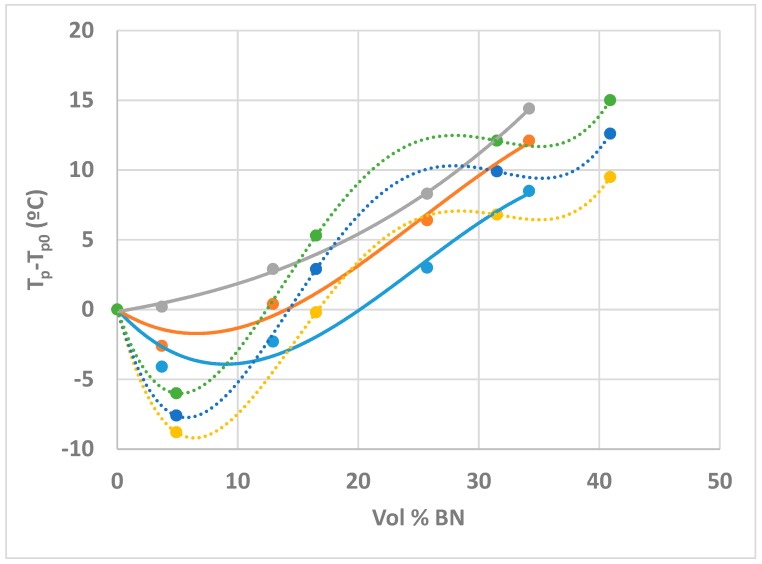
Plot of *T*_p_ − *T*_p0_ as a function of vol % BN for the systems ETLBN80 and ETLBN80/6, respectively, at the heating rates of 2 (dark blue, purple), 5 (red, light blue), and 10 K/min (green, orange). The curves are drawn to guide the eye: full lines for ETLBN80, dotted lines for ETLBN80/6.

In Figure 6, it can be seen that the 80/6 hybrid system displays the same dependence of the peak exotherm temperature on BN content as was observed for the simple system with 80 µm agglomerates and shown in Figure 4. A direct comparison of the two systems is shown in Figure 8; very similar results were obtained for both hybrid systems, ETLBN80/6 and ETLBN80/2. The most noticeable difference between the simple system and the hybrid system is evident at small BN contents, where the addition of the smaller BN particles significantly accelerates the reaction, advancing the peak temperature by about 5 °C. It appears that adding the smaller particles to fill, at least partly, the spaces between the larger particles results in a better connectivity between the BN particles, and hence an improved thermal conductivity, which advances the reaction.

For higher BN contents, the situation is more complicated, because there is now the competing effect of improved dissipation of the exothermic heat generated by the reaction, which dominates the overall heat transfer and results in a retardation of the reaction. Although the trend lines drawn to guide the eye in Figure 8 are rather different for the simple and hybrid systems, this is not intended to imply that there are significant differences between them for BN contents greater than 10 vol %. In fact, in this range of BN contents greater than 10 vol %, it could be argued that the two systems display the same behaviour at each of the three different heating rates; the difference between them is simply that the hybrid results are at higher volume percentages as a consequence of the composition of the samples, as defined in Table 1.

The remarkable behaviour of the samples with 10 vol % BN is even more pronounced in the isothermal cure shown in Figure 7. Here, the peak exotherm heat flow is even higher for the 10 vol % sample than it is for the sample without filler, and is dramatically displaced to shorter times. The effect of the addition of the smaller particles in these hybrids is also evident at short times in the ETLBN80/2-50 and ETLBN80/2-60 samples in Figure 7, where there appears to be an initial reaction which is then followed by the main reaction. These are not, however, two different chemical reactions, as the network structure of the cured system, characterised by the heat of reaction and *T*_g∞_, is independent of BN content; this effect is simply a consequence of heat transfer.

### 3.2. Density Measurements

The densities of the various samples, determined by Archimedes principle, are collected in Table 4. The vol % BN is determined from the measured density by assuming a density of 2.1 g/cm^3^ for the BN particles; the results are included in Table 4, and are compared with the approximate values given earlier in Table 1, which were calculated from the compositions of the samples by weight and the densities of the individual components. In all cases except one (ETLBN80/2-60), the measured vol % BN is between 2% and 5% higher than the calculated value; this is consistent with a shrinkage on cure, which is typically between 2% and 7% in volume [43,44]. The measured vol % BN for sample ETLBN80/2-60 is lower than expected, possibly indicative of the presence of some air bubbles, which would result in a decreased value of the thermal conductivity for this sample. The thermal conductivity measurements are discussed immediately below, but comparison of the values given in Table 4 for ETLBN80/2-60 and ETLBN80/6-60, where it can be seen that the thermal conductivity of the former is about 4% lower than the latter, suggests that there may well have been some air bubbles in the 80/2 hybrid sample. 

### 3.3. Thermal Conductivity Measurements

**Figure 9:**
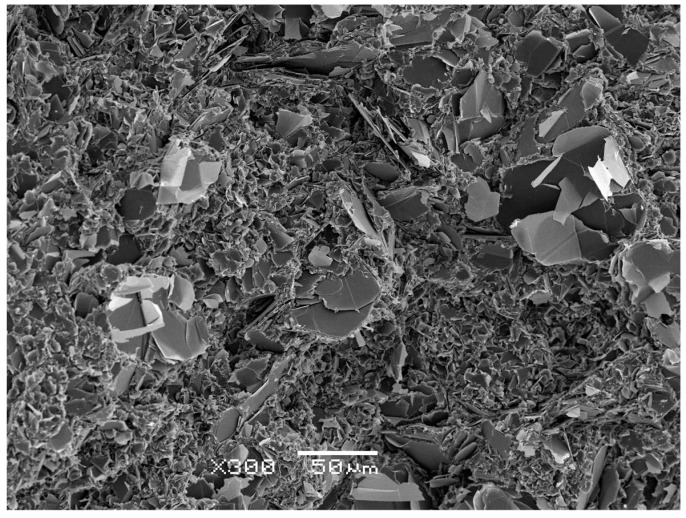
Scanning electron micrograph of epoxy-BN hybrid system ETLBN80/2-50. Scale bar is 50 µm.

Before reporting the thermal conductivity measurements, it is interesting to examine the dispersion of the filler particles in the cured epoxy matrix. Scanning electron micrographs were made of the fracture surfaces of the cured composite systems, and a typical example is shown in Figure 9 for the hybrid sample ETLBN80/2-50 at a magnification of 300×. It can be seen that the dispersion is good, with the larger 80 µm agglomerates surrounded by the smaller 2 µm platelets. This quality of dispersion was observed in all the epoxy-BN systems studied here.

**Figure 10:**
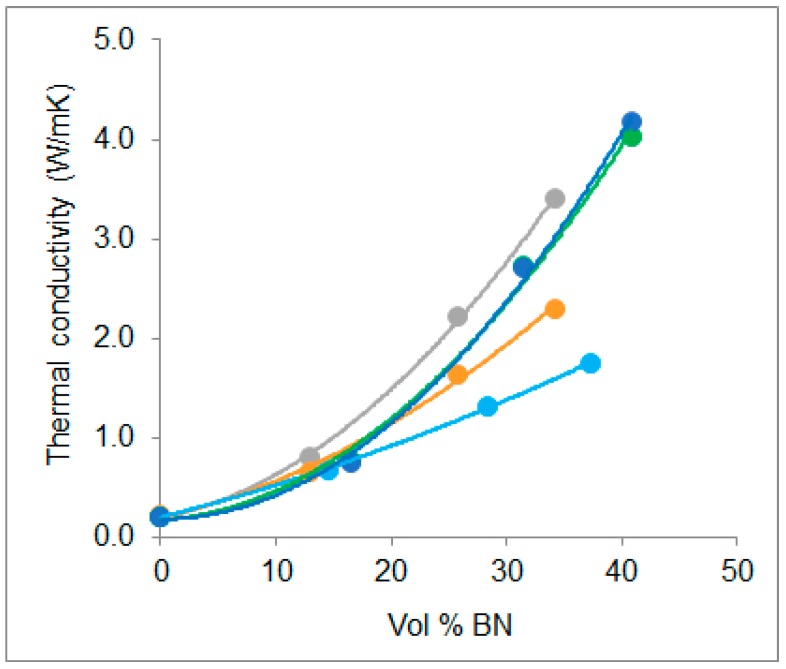
Plot of thermal conductivity of epoxy-BN systems as a function of vol % BN: epoxy-diamine, light blue; ETLBN6, orange; ETLBN80, grey; ETLBN80/2, green; ETLBN80/6, dark blue.

The thermal conductivities, λ, of the various samples, measured by the transient hot bridge method, are presented in Table 4 and plotted as a function of the calculated vol % BN in Figure 10. Also included in this figure, for comparison, are our earlier results obtained with 6 µm BN particles incorporated in two different epoxy matrix systems: epoxy-thiol, as used in the present work, and epoxy-diamine [19]. This allows us to investigate the effect on the thermal conductivity of different BN particle sizes and of different epoxy matrix systems.

The first observation that can be made is that, as expected, the thermal conductivity increases with BN content, and the dependence follows the trend discussed in the introduction and illustrated in Figure 1. Comparing the conductivities of the epoxy-diamine system and the epoxy-thiol system (ETLBN6), both systems being filled with 6 µm BN particles, shown in Figure 10, the latter clearly has a higher conductivity when the BN content exceeds about 10 vol %. It is interesting to compare these results with the respective cure kinetics for these two systems. For the epoxy-diamine system, investigated in earlier work [19], there was no significant dependence of the peak temperature, *T*_p_, in non-isothermal cure or of the time, *t*_p_, for the peak exotherm in isothermal cure, on the BN content. By contrast, both of these characteristic parameters of the cure kinetics showed a systematic dependence on BN content for the ETLBN6 system, as was shown in Figure 3 and Figure 5 for non-isothermal and isothermal cure, respectively. The interpretation of this systematic dependence for the ETLBN6 system was that it was a consequence of the improved heat transfer in the curing sample, which would correspond to a higher thermal conductivity in this system in comparison with the epoxy-diamine, and this is exactly what is demonstrated in Figure 10. The improved thermal conductivity in the epoxy-thiol system is believed to be a consequence of a better interface between the epoxy matrix and the BN particle surfaces resulting from a Lewis acid-base interaction between the sulphur of the thiol and the boron, this interaction not being present in the epoxy-diamine system.

Likewise, the cure kinetics of the epoxy-thiol system with 80 µm BN particles also shows a systematic dependence on BN content, in which the peak temperature in non-isothermal cure and the peak time in isothermal cure both decrease and then increase as the BN content increases, as was shown in Figure 3 and Figure 5, respectively. For the ETLBN80 system, though, the initial decrease occurs at lower BN contents than for the ETLBN6 system, and the subsequent increase is more marked than that for the ETLBN6 system at higher BN contents. If, as we suggest above, this dependence is related to the effects of thermal conductivity, then one might anticipate a greater thermal conductivity in the composites with 80 µm particles, and this is indeed what is observed in the results shown in Figure 10. Interestingly, though, in our earlier work [19], we observed that when aluminium nitride (AlN) rather than BN is used as the filler, the initial decrease in the peak temperature during non-isothermal cure is significantly greater than that for BN. This suggests a higher thermal conductivity for the epoxy-AlN composites, and yet the measured thermal conductivity of the cured epoxy-AlN samples is in fact lower than that for the epoxy-BN composites at the same filler content. This is almost certainly a consequence of a poorer interface in the cured epoxy-AlN composites; the change in the interfacial behaviour between the sample during cure (liquid) and the fully cured sample (solid) plays an important role in this respect. 

Furthermore, when hybrid samples are fabricated, either the 80/6 or 80/2 combination, the thermal conductivity decreases with respect to the simple mixtures with 80 µm particles, for the same vol % BN, as would be expected if the smaller particles result in a smaller increase in the thermal conductivity. On the other hand, the hybrid samples provide the advantage that a more tractable material in the uncured state can be achieved, thus allowing higher BN contents, and hence higher thermal conductivities, as shown by the results for these two hybrids presented in Figure 10, where a thermal conductivity greater than 4.0 W/mK is attained.

These values of thermal conductivity presented in Figure 10 for the simple and hybrid samples with 80 µm BN particles can be compared, with the summary of values extracted from the literature and shown in Figure 1, where it can be seen that they fall above nearly all the other values, and quite close to the upper trend line. This reinforces our hypothesis that the interaction between the epoxy-thiol system and the BN particles plays an important role in improving the interface between matrix and filler. It is pertinent, therefore, also to examine the conditions under which higher values of thermal conductivity included in Figure 1 have been obtained in the range of 10 to 40 vol % BN; in particular, three sets of results are worthy of consideration.

Gaska et al. [10] use a DGEBA epoxy similar to ours, but cured with an anhydride rather than thiol, and the filler particles are either 13 µm hexagonal platelets or 30 µm agglomerates, approximately spherical. The specific surface areas of these particles are, respectively, 5.5 and 7.0 m^2^/g, and the surfaces are not treated in any way. The thermal conductivity is measured under high vacuum at room temperature. The slightly higher values of thermal conductivity found for the samples made with 30 µm BN particles is in agreement with the usually observed increase in thermal conductivity with increasing BN particle size. On the other hand, the reason why Gaska et al. [10] obtain higher thermal conductivities than most other workers, their results following closely the upper trend curve in Figure 1, is unclear. It is unlikely to be a consequence of the use of an anhydride curing agent, as this is also the system used for the majority of the results presented in Figure 1 [6,9,10,11,12,13,14,16,17,18,25,26,27,28,35,36,39], for which there is no clear trend.

Jang et al. [20] use an unspecified epoxy resin cured with a dicyandiamide, filled with 5 to 10 µm hexagonal BN platelets. The vol % BN in Figure 1 is estimated from the weights of each component in the preparation procedure. When the BN particles are surface treated with a silane coupling agent, either propyl trimethoxysilane or hexamethyl trimethoxysilane, by a sol-gel process, the thermal conductivity is found to increase from 2.40 W/mK for the untreated epoxy-BN composite to 3.37 and 3.49 W/mK, respectively. The value for the untreated sample is very close to our value for the 80/2 and 80/6 hybrids at the same vol % BN (Figure 10), whereas, as is often but not universally observed, the effect of surface treatment is to increase the thermal conductivity, in this case significantly. The use of a coupling agent or other surface treatment in our own samples is clearly of interest, and is currently under investigation.

Kim et al. [23] also use an unspecified epoxy resin, filled with BN particles of different morphologies: nearly spherical agglomerates (A-BN, average diameter 50 µm) and plate-like whiskers (W-BN, average diameter 10 µm and length 30 µm). The BN particles were treated by suspension in sodium hydroxide, in order to introduce hydroxyl groups onto the surface. Composites with different compositions of A-BN and W-BN had BN contents ranging from 17 to 43 vol % but, while the thermal conductivity increased with BN content, there was no obvious difference between the different BN morphologies. Nevertheless, the variation of thermal conductivity with BN content is noticeably different from the majority of results in Figure 1, with values higher than the upper trend curve at low BN contents, and values significantly lower than the upper trend curve at higher BN contents, which may be a consequence of the different morphologies in these samples. In our own epoxy-thiol system filled with BN particles, the effect of filler particle morphology is currently being studied by comparing the cure kinetics and thermal conductivities of both BN platelets and agglomerates.

## 4. Conclusions

The cure kinetics of epoxy-BN composites, in which the epoxy is cured with a thiol, has been studied by DSC, and has been found to be correlated with the thermal conductivity of the cured composites. This is the first time that both the cure kinetics and thermal conductivity measurements have been reported for the same system, and the correlation has been shown to have significant consequences with respect to the use of epoxy-thiol as the matrix material in composites for high thermal conductivity. The most important aspects can be summarised as follows:

- The heat of reaction in both non-isothermal and isothermal cure is independent of the BN content, as is also the glass transition temperature of the fully cured samples, indicating that the network structure of the epoxy-thiol matrix is not influenced by the presence of BN particles.
- On the other hand, the exothermic curing reaction has a maximum heat flow that occurs at a temperature (for non-isothermal cure) or a time (for isothermal cure) which depends systematically on the BN content, first decreasing at low BN contents, and then increasing as the BN content increases.
- This behaviour is attributed to the effects of heat transfer both into (before the exothermic reaction) and out of (after the exothermic reaction) the sample, the effect being more marked for the epoxy-BN composites with 80 µm particles than it is for those with 6 µm particles.
- The dependence of the cure reaction kinetics on BN content is not observed for epoxy-BN composites in which the epoxy is cured with a diamine, from which it is concluded that the Lewis acid-base interaction between the thiol and the BN particles results in an improved matrix-filler interface.
- The fully cured epoxy-BN composites filled with 80 µm particles, for which the systematic effect of BN on the cure kinetics was more pronounced, have a higher thermal conductivity than do the composites filled with 6 µm particles, which correlates with the DSC observations.
- The thermal conductivity for all the composites increases with BN content, and for a given BN vol %, the values obtained in this work are higher than most others in the literature.
- The use of hybrid samples, in which BN particles of two different sizes, 80 and 6 µm, are combined, allows a greater vol % BN to be achieved, and results in a thermal conductivity of over 4.0 W/mK for a BN content of 42 vol %.

## Figures and Tables

**Table 1:** Composition of samples: parts by mass, wt % BN and the approximate vol % BN.

Samples	Parts by mass					wt % BN	vol % BN calculated
Simple mixtures	Epoxy	BN filler, 80 µm		Thiol	LC-80		
ETL	100.0	0		66.7	2.0	0	0
ETLBN80-10	90.0	10.0		60.0	1.8	6.2	3.7
ETLBN80-30	70.0	30.0		46.7	1.4	20.3	12.9
ETLBN80-50	50.0	50.0		33.3	1.0	37.2	25.7
ETLBN80-60	40.0	60.0		26.7	0.8	47.1	34.2
80/6 hybrids		80 µm	6 µm				
ETLBN80/6-10	90.0	10.0	3.3	60.0	1.8	8.1	4.9
ETLBN80/6-30	70.0	30.0	10.0	46.7	1.4	25.3	16.5
ETLBN80/6-50	50.0	50.0	16.7	33.3	1.0	44.2	31.6
ETLBN80/6-60	40.0	60.0	20.0	26.7	0.8	54.2	40.9
80/2 hybrids		80 µm	2 µm				
ETLBN80/2-10	90.0	10.0	3.3	60.0	1.8	8.1	4.9
ETLBN80/2-30	70.0	30.0	10.0	46.7	1.4	25.3	16.5
ETLBN80/2-50	50.0	50.0	16.7	33.3	1.0	44.2	31.6
ETLBN80/2-60	40.0	60.0	20.0	26.7	0.8	54.2	40.9

**Table 2:** Results for ETLBN80 system from non-isothermal DSC cure experiments at different heating rates, β: *T*_g0_, Δ*H*, *T*_g∞_, *T*_p_.

Sample	β (K/min)	*T*_g0_ (°C)	∆*H* (J/g)	∆*H* (kJ/ee)	*T*_g∞_ (°C)	*T*_p_ (°C)
**ETL**	2	−37.3	425	131	53.8	83.8
	5	−37.7	420	129	53.0	93.9
	10	−37.2	416	128	52.5	102.7
**ETLBN80-10**	2	−37.9	403	132	55.8	79.7
	5	−36.9	404	133	55.0	91.3
	10	−35.4	397	131	54.7	102.9
**ETLBN80-30**	2	−38.1	334	128	54.1	81.5
	5	−36.9	333	128	52.9	94.3
	10	−35.6	334	129	52.8	105.6
**ETLBN80-50**	2	−38.4	267	131	54.7	86.7
	5	−37.4	264	130	54.8	100.3
	10	−36.7	260	128	53.9	111.0
**ETLBN80-60**	2	−38.8	228	132	54.8	92.3
	5	−37.7	220	128	54.8	105.9
	10	−36.2	220	128	54.5	117.1

**Table 3:** Results for ETLBN80 system from isothermal DSC cure experiments at different cure temperatures, *T*_c_: Δ*H*, *T*_g∞_, *t*_p_.

Sample	*T*_c_ (°C)	∆*H* (J/g)	∆*H* (kJ/ee)	*T*_g∞_ (°C)	*t*_p_ (min)
**ETL**	60	431	132	53.6	58
	70	399	122	54.7	11
	80	430	132	53.3	3.7
**ETLBN80-10**	60	401	131	52.8	20
	70	396	130	53.4	9.0
	80	398	131	52.7	3.8
**ETLBN80-30**	60	329	126	52.0	24
	70	344	133	53.7	11
	80	329	127	52.6	4.8
**ETLBN80-50**	60	257	126	52.6	41
	70	256	126	53.6	17
	80	260	128	51.6	7.5
**ETLBN80-60**	60	212	123	52.3	62
	70	221	129	53.0	27
	80	206	120	51.7	13

**Table 4:** Densities and thermal conductivities of samples studied, as a function of vol % BN.

Sample	Density (g/cm^3^)	vol % BN measured	vol % BN calculated	λ (W/mK)
ETL		0.0	0.0	0.20
ETLBN80-30	1.39	13.4	12.9	0.80
ETLBN80-50	1.51	26.7	25.7	2.21
ETLBN80-60	1.56	35.0	34.2	3.40
ETLBN80/2-30	1.42	17.1	16.5	0.77
ETLBN80/2-50	1.54	32.4	31.5	2.72
ETLBN80/2-60	1.58	40.8	40.9	4.02
ETLBN80/6-30	1.42	17.1	16.5	0.75
ETLBN80/6-50	1.57	33.0	31.5	2.71
ETLBN80/6-60	1.63	42.1	40.9	4.17
